# Functional Muffins Exert Bifidogenic Effects along with Highly Product-Specific Effects on the Human Gut Microbiota Ex Vivo

**DOI:** 10.3390/metabo14090497

**Published:** 2024-09-14

**Authors:** Stef Deyaert, Jonas Poppe, Lam Dai Vu, Aurélien Baudot, Sarah Bubeck, Thomas Bayne, Kiran Krishnan, Morgan Giusto, Samuel Moltz, Pieter Van den Abbeele

**Affiliations:** 1 Cryptobiotix, Technologiepark-Zwijnaarde 82, 9052 Ghent, Belgium; stef.deyaert@cryptobiotix.com (S.D.);; 2 Bubeck Scientific Communications, 194 Rainbow Drive #9418, Livingston, TX 77399, USA; 3 Microbiome Labs, 101 E Town Pl, Saint Augustine, FL 92092, USA; 4 Novonesis, Biologiens Vej 2, 2800 Lyngby, Denmark

**Keywords:** systemic intestinal fermentation research (SIFR), berry blast muffin (BBM), lemon chia muffin (LCM), oat spice mookie (OSM), short-chain fatty acid (SCFA), functional foods, probiotics, prebiotics

## Abstract

GoodBiome™ Foods are functional foods containing a probiotic (*Bacillus subtilis* HU58™) and prebiotics (mainly inulin). Their effects on the human gut microbiota were assessed using ex vivo SIFR^®^ technology, which has been validated to provide clinically predictive insights. GoodBiome™ Foods (BBM/LCM/OSM) were subjected to oral, gastric, and small intestinal digestion/absorption, after which their impact on the gut microbiome of four adults was assessed (n = 3). All GoodBiome™ Foods boosted health-related SCFA acetate (+13.1/14.1/13.8 mM for BBM/LCM/OSM), propionate (particularly OSM; +7.4/7.5/8.9 mM for BBM/LCM/OSM) and butyrate (particularly BBM; +2.6/2.1/1.4 mM for BBM/LCM/OSM). This is related to the increase in Bifidobacterium species (*B. catenulatum*, *B. adolescentis*, *B. pseudocatenulatum*), *Coprococcus catus* and Bacteroidetes members (*Bacteroides caccae*, *Phocaeicola dorei*, *P. massiliensis*), likely mediated via inulin. Further, the potent propionogenic potential of OSM related to increased Bacteroidetes members known to ferment oats (s key ingredient of OSM), while the butyrogenic potential of BBM related to a specific increase in Anaerobutyricum *hallii*, a butyrate producer specialized in the fermentation of erythritol (key ingredient of BBM). In addition, OSM/BBM suppressed the pathogen *Clostridioides difficile*, potentially due to inclusion of HU58™ in GoodBiome™ Foods. Finally, all products enhanced a spectrum of metabolites well beyond SCFA, including vitamins (B3/B6), essential amino acids, and health-related metabolites such as indole-3-propionic acid. Overall, the addition of specific ingredients to complex foods was shown to specifically modulate the gut microbiome, potentially contributing to health benefits. Noticeably, our findings contradict a recent in vitro study, underscoring the critical role of employing a physiologically relevant digestion/absorption procedure for a more accurate evaluation of the microbiome-modulating potential of complex foods.

## 1. Introduction

The gut microbiome has important effects on the human host, including the ability to impact the host immune response and metabolism [1,2,3,4,5]. It is mainly is composed of six phyla including mainly *Firmicutes*, *Bacteroidetes*, and at lower abundances, amongst others, also *Actinobacteria*, *Proteobacteria*, and *Verrucomicrobia* [6]. Gut microbiome dysbiosis (i.e., an imbalance in the gut microbial community which alters normal microbiome activity) is associated with several intestinal disorders, including irritable bowel syndrome, inflammatory bowel disease, and celiac disease, as well as other disorders such as asthma, cardiovascular disease, metabolic syndrome, allergy, and obesity [7]. Diet can strongly affect the composition and function of the gut microbiota [8]. In fact, a healthy diet (i.e., one that avoids Western dietary components and is high in fiber, high-quality protein, and micronutrients) is thought to protect against dysbiosis [9]. Fermentable fibers and non-digestible polysaccharides are particularly important for healthy gut microbiome function as they support the growth of beneficial microbes and are fermented into short-chain fatty acids (SCFAs) [10] which have numerous benefits to the host [11,12]. These benefits include acting as an energy source for intestinal epithelial cells, strengthening the intestinal barrier, and promoting colonic blood flow [11,12].

Functional foods, which are defined as ‘foods that provide additional health benefits that may reduce disease risk and/or promote health’ [13], have been viewed with increasing interest in recent years. Examples of functional foods include kimchi, yogurt, and oats. These foods are often rich in probiotics and/or prebiotics, which are thought to provide the additional health benefits associated with functional foods. GoodBiome™ Foods are a collection of functional foods that contain both the probiotic *Bacillus subtilis* HU58™ and prebiotics (e.g., inulin). Inulin is a polymer of β(2,1) bond-linked fructose residues with a chain-terminating glucose, with native inulin typically having a degree of polymerization between 3 and 60 [14]. Inulin is known to beneficially impact the gut microbiome and host health [15]. *Bacillus* spp. are spore-forming bacteria that have an advantage over non-spore-forming probiotics in that they are resistant to heat, desiccation, and pH fluctuations [16]. There is growing evidence that *Bacillus* spp. have the ability to impart health benefits to the host, such as a reduced occurrence of diarrhea associated with antibiotic use [17], improvements in acute diarrhea [18], immune effects in healthy individuals [17,19], and reduced pain, discomfort, and bloating in those with irritable bowel syndrome [20,21,22]. The probiotic strain *B. subtilis* HU58™ has been shown to provide health benefits to both animals and humans [23,24,25,26].

The ex vivo SIFR^®^ technology (Systemic Intestinal Fermentation Research) is an automated technology that simulates colonic fermentation [27], while an advanced digestion and simulation of small intestinal absorption can also be integrated for products that contain digestible carbohydrates, proteins, and/or lipids [28]. The technology has been optimized to minimize the bias in microbiota composition between the in vivo-derived microbiota and the microbiota that colonizes the bioreactors during SIFR^®^ studies, hence classifying studies with the technology as ex vivo studies [27]. As recently published by Van den Abbeele et al. [27], the SIFR^®^ technology has been validated by studying the impact of three structurally different carbohydrates (inulin, 2′fucosyl-lactose, and resistant dextrin). It followed that changes observed in the SIFR^®^ technology within 24–48 h corresponded with in vivo observations upon the repeated daily intake of the aforementioned carbohydrates over weeks (2–6 weeks), down to the species level. Applications of the SIFR^®^ technology meanwhile go well beyond prebiotics and range from characterizing the microbiome-modulating potential of probiotics, synbiotics [29], and sweeteners [30], to studying the development of age-specific ingredients [31], the investigation of fiber specificity [32], or the assessment of microbial diversity (using novel indices) [33], and even evaluating the impact on intestinal barrier integrity and/or immune functioning [28].

During this study, we applied the validated ex vivo SIFR^®^ technology to investigate the gut microbiome-modulating potential of GoodBiome™ Foods. Given that GoodBiome™ Foods contain digestible carbohydrates and proteins, a critical aspect was to apply a physiologically relevant simulation of digestion and absorption before the colonic incubations [28].

## 2. Materials and Methods

### 2.1. GoodBiome™ Foods

Three GoodBiome™ Foods, the Berry Blast Muffin (BBM), the Lemon Chia Muffin (LCM), and the Oat Spice Mookie (OSM), were evaluated (Microbiome Labs; Glenview, IL, USA). Each of these products contains prebiotics, including inulin powder (10 g per serving of 50 g), and the probiotic strain *B. subtillis* HU58™ (10^9^ CFU per serving of 50 g). The full list of ingredients for each product is listed in Appendix A. Each GoodBiome™ Foods product was provided as a 50 g powder (single serving) and was prepared according to the manufacturer’s instructions. Briefly, water was added to the powder (BBM and LCM, 59 mL water; OSM, 44 mL water), mixed until well combined, and cooked in the microwave (R-941-STW, Sharp, Mechelen, Belgium) (BBM and LCM, 1 min 30 s at 1050 W; OSM, 2 min at 1050 W). A blank reactor (i.e., no food product) was used as the unsupplemented control (no substrate control, NSC).

### 2.2. Experimental Design, Timeline, and Analysis

**Figure 1:**
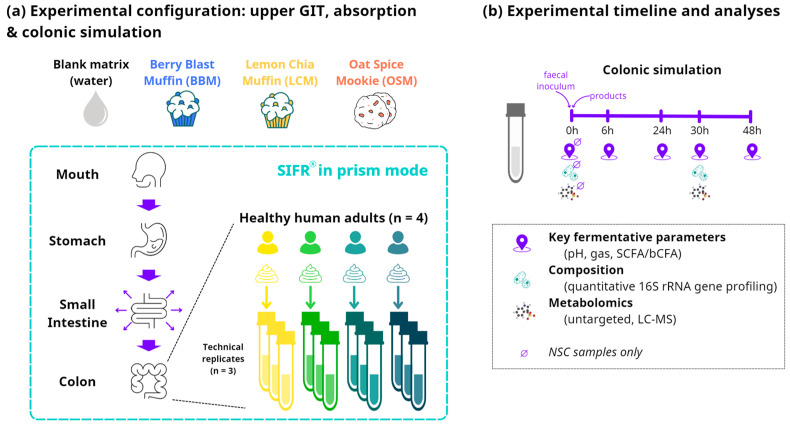
**Schematic overview of the study design using ex vivo SIFR^®^ technology.** (**a**) Reactor design using the ex vivo SIFR^®^ technology to evaluate the impact of GoodBiome^TM^ Foods against an unsupplemented parallel control (NSC = no substrate control). (**b**) Timeline and analysis at different timepoints.

The upper gastrointestinal digestion and colonic fermentation of the GoodBiome™ Foods were investigated using the SIFR^®^ technology (Figure 1a). Oral, gastric, and small intestine digestion were simulated as recently described [27,34]. Briefly, test products (or distilled H_2_O for NSC) were subjected to oral, gastric, and small intestinal digestion according to the INFOGEST 2.0 consensus method published by Brodkorb et al. [35]. To make the digestion method compatible with subsequent colonic fermentation, oxygen was removed and small intestinal absorption was simulated using dialysis membranes, as described recently [34]. Together, these methods facilitated the simulation of the upper gastrointestinal tract and enabled coupling with subsequent colonic incubations. 

At the start of the colonic incubations, individual fecal samples were processed in a bioreactor management device (Cryptobiotix, Ghent, Belgium) [27]. For the colonic incubations, individual fecal samples from four healthy adults were incubated (n = 3 per donor) with the digested test products. Each test product was paired with each donor and separate reactors were run in parallel for each timepoint to avoid interference of sampling. For each of the four fecal microbiota, an unsupplemented control (NSC) was initiated simultaneously, consisting of background medium and microbiota without a test product. The advantage of comparing test products to an NSC is that any changes observed between the NSC and test products can solely be attributed to the addition of the test products. Upon gas pressure measurement in the headspace, liquid samples were collected from the colonic reactors and analyzed at five timepoints: 0 h, 6 h, 24 h, 30 h, and 48 h (Figure 1b). Key fermentation parameters, including the measurement of pH, gas production, SCFAs, and branched-chain fatty acids (bCFAs), were assessed at all five timepoints. Metagenomics and metabolomics analyses were performed on samples collected at 0 h and 30 h. 

Fecal samples were collected in accordance with a protocol approved by the Ethics Committee of the University Hospital Ghent (reference no. BC-09977). All donors provided written informed consent to the collection and use of their fecal samples.

### 2.3. Key Fermentation Parameters 

The acidification of the colonic medium is a measure for the degree of bacterial activity. The pH was measured using an electrode (Hannah Instruments Edge HI2002, Temse, Belgium). As SIFR^®^ incubations are performed in closed reactors, one can determine gas accumulation in the headspace by penetrating the rubber septum with a needle connected to a pressure meter. Concentrations of the SCFAs acetate, propionate, butyrate, and valerate, and bCFAs (combined concentration of isobutyrate, isovalerate, and isocaproate) were determined using gas chromatography with flame-ionization detection (Trace 1300, Thermo Fisher Scientific, Merelbeke, Belgium) upon diethyl ether extraction, as previously described [32].

### 2.4. Microbial Community Composition

Quantitative shallow shotgun sequencing was performed on colonic samples collected at 0 h and 30 h. Quantitative data were obtained by correcting abundances (%; shallow shotgun sequencing) with total cell counts for each sample (cells/mL; flow cytometry), resulting in estimated cell counts/mL of different taxonomic groups, overall allowing to obtain more representative insights in the impact of interventions on the gut microbiota.

First, a bacterial cell pellet was obtained via the centrifugation of a 1 mL sample during 5 min at 9000× *g*. DNA was extracted via the SPINeasy DNA Kit for Soil (MP Biomedicals, Eschwege, Germany), according to manufacturer’s instructions. Following DNA extraction, a library was prepared using the Nextera XT DNA Library Preparation Kit (Illumina, San Diego, CA, USA) and IDT Unique Dual Indexes (total DNA input, 1 ng). A proportional amount of Illumina Nextera XT fragmentation enzyme was added to fragment genomic DNA. Libraries were constructed, purified, and quantified as previously described [27], then sequenced on an Illumina Nextseq 2000 platform 2 × 150 base pairs. The CosmosID-HUB Microbiome Platform (CosmosID Inc., Germantown, MD, USA) was used to convert unassembled sequencing reads to relative abundances (%) [36,37]. For total cell count analysis, liquid samples were diluted in anaerobic phosphate-buffered saline, stained with SYTO 16 (1 μM), and counted using a BD FACS Verse flow cytometer (BD, Aalst, Belgium). Data were analyzed using FlowJo, version 10.8.1. 

### 2.5. Metabolomics

Untargeted ultra-performance liquid chromatography with tandem mass spectrometry was performed at 0 h (n = 1 per donor) and 30 h (n = 3 per donor). A Vanquish UHPLC (Thermo Scientific, Germering, Germany) coupled to a Orbitrap Exploris 240 MS (Thermo Scientific, Bremen, Germany) with an electrospray ionization interface as the ionization source (applied in both negative and positive ionization mode) was used to carry out the UPLC-MS/MS experiments. UPLC-MS/MS was performed according to a slightly modified version of the protocol described by Doneanu et al. [38]. Peak areas were extracted using Compound Discoverer 3.1 (ThermoFisher Scientific) as well as a manual extraction of compounds using Skyline 21.1 (MacCoss Lab Software, University of Washington, Seattle, WA, USA) [39], which included an in-house library. Compound identification was performed at three levels: level 1 (retention times (compared against in-house authentic standards), accurate mass (with an accepted deviation of 3 ppm), and MS/MS spectra)), level 2a (retention times and accurate mass), level 2b (accurate mass and MS/MS spectra), and level 3 (accurate mass alone).

### 2.6. Statistical Analysis

All univariate and multivariate analyses were performed using R (version 4.4.0; www.r-project.org; accessed on 26 July 2024). The significance of the supplementation effects compared with the NSC were assessed via repeated measure ANOVA analyses (based on paired testing among the 6 human adults) using the rstatix package, with *p*-value correction according to Benjamini–Hochberg [40,41]. 

For the analysis of microbial composition, different measures were taken. First, the statistical analysis was performed on the log_2_-transformed values. Second, a value of a given taxonomic group below the limit of detection (LOD) was considered equal to the overall LOD according to the procedure elaborated by Van den Abbeele et al. (2023) [27]. Third, a threshold was set to retain the 100 most abundant species in the analysis to avoid excessive *p*-value corrections. Finally, taxa that were not significantly affected were further assessed for consistent changes. To be considered as consistently increasing/decreasing for either treatment, taxa had to be present in at least two out of four test subjects and consistently increasing or decreasing for all the test subjects where the taxa were detected. Statistical analysis for metabolomics was performed only on level 1 and 2a metabolites. For the analysis of the metabolites, only metabolites produced along the incubations were considered. Metabolites were considered produced if their concentration increased in at least one of the treatments at the final timepoint for at least two out of four test subjects. All visualizations in R were enhanced using the ggplot2 package [42].

## 3. Results

### 3.1. The Study Cohort Covered Enterotypic Differences Described for Human Adult Gut Microbiota

**Figure 2:**
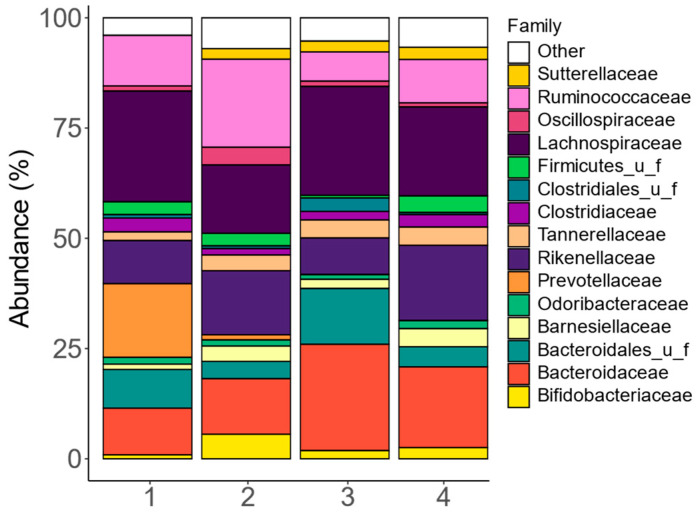
**The fecal microbiota covered clinically relevant interpersonal differences.** Abundances (%) of the key families (top 15), as quantified via shallow shotgun sequencing, in the fecal microbiota of each of the four human adults that provided a fecal donation for the current SIFR^®^ study.

At the family level, the fecal microbiota composition among the four human adults exhibited notable interpersonal differences, mostly due to differences in *Bacteroidaceae*, *Bacteroidales_u_f*, *Ruminococcaceae*, *Lachnospiraceae*, and *Prevotellaceae* (Figure 2 and Figure S1). The stratification of fecal microbiota based on these families is in line with the classification of fecal microbiota according to the concept of enterotypes [43].

### 3.2. GoodBiome™ Foods Stimulated the Metabolic Activity of the Gut Microbiota and the Production of Short-Chain Fatty Acids

**Figure 3:**
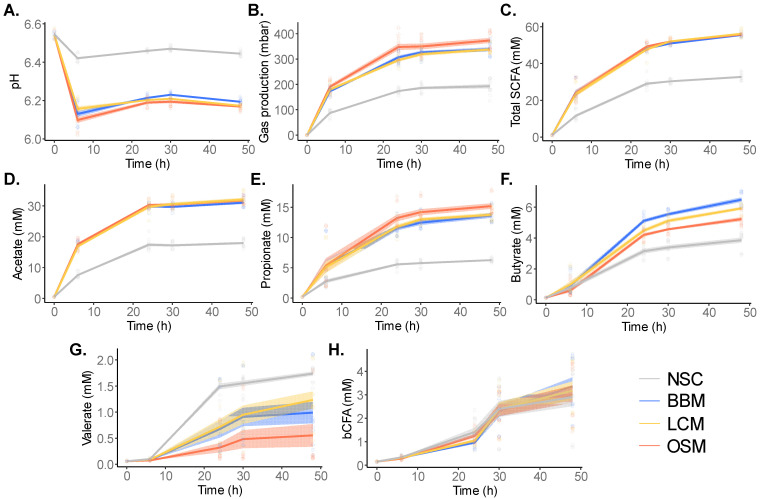
**GoodBiome™ Foods exerted marked effects on microbial metabolic activity over time.** The effects on (**A**) pH, (**B**) gas production, (**C**) total SCFA, (**D**) acetate, (**E**) propionate, (**F**) butyrate, (**G**) valerate, and (**H**) bCFA were compared for GoodBiome™ Foods versus an unsupplemented control (NSC) at 6 h, 24 h, 30 h, and 48 h after the initiation of colonic incubation. Data were presented as means across simulations for four individual donors (n = 3 per donor). The statistical significance of the treatment effects for the test products vs. NSC within each timepoint can be found in Figures S2 and S3.

To assess the product-specific effects on metabolic activity of the gut microbiota, key fermentation parameters were recorded at 0 h, 6 h, 24 h, 30 h, and 48 h after the initiation of the SIFR^®^ colonic fermentation (Figure 3, Figures S2 and S3).

At the timepoint of in-depth analysis (30 h), coefficients of variation (CV = standard deviation/average) across technical replicates were on average as low as 1.1% for SCFA levels. This covered variation due to reactor preparation, incubation, and sampling, but also the technical variation of SCFA analysis itself (diethyl ether extraction and subsequent analysis via GC-FID). Such high technical reproducibility renders the SIFR^®^ technology very sensitive in identifying small but significant changes.

All GoodBiome™ Foods significantly decreased pH and increased gas production relative to NSC at all timepoints (Figure 3A,B), indicating increased metabolic activity due to the fermentation of the test products.

Further, the fermentation of the GoodBiome™ Foods also markedly impacted SCFA and bCFA production (Figure 3C–H). This was evidenced by the increased production of acetate, propionate, and butyrate with all GoodBiome™ Foods versus the NSC at all timepoints (Figure 3D–F and Figure S3). While similar acetate levels were observed for all three test products (Figure 3D and Figure S3A), propionate production was notably higher with OSM (Figure 3E and Figure S3B), while butyrate was more enhanced by LCM and especially BBM at 24 h, 30 h, and 48 h (Figure 3F and Figure S3C). Overall, this resulted in similar total SCFAs with all three GoodBiome™ Foods (Figure 3C and Figure S2C). The only SCFA that was not boosted by GoodBiome™ Foods was valerate which significantly decreased, especially with OSM at 24 h, 30 h, and 48 h (Figure 3G and Figure S3D).

Finally, while SCFA production mostly occurred between 0 and 24 h (Figure 3C), bCFA production mostly occurred between 24 and 48 h (Figure 3H). This suggests that saccharolytic fermentation mainly occurred between 0 and 24 h, with additional proteolytic activity occurring between 24 and 48 h. In contrast to their potent effects on SCFA, GoodBiome™ Foods did not impact bCFA (Figure 3H and Figure S2D). Given that the saccharolytic gut microbes were mostly active during the 0–24 h time frame, their cells could potentially start to lyse between 24 and 48 h. Therefore, aiming to cover both saccharolytic and proteolytic gut microbes, the 30 h timepoint was selected for an in-depth analysis of microbial composition and metabolite production.

### 3.3. Fermentation of GoodBiome™ Foods Resulted in Product-Specific Changes in the Composition of Gut Microbial Community

The bacterial cell density increased between 0 h and 30 h for all colonic incubations. Additionally, the fermentation of the Goodbiome™ Foods further enhanced total cell growth compared to the NSC (Figure S4A). Although microbial diversity remained high in the test product incubations, it was significantly lower versus NSC as measured by three diversity indices: the Chao diversity index, Reciprocal Simpson diversity index, and Shannon diversity index (Figure S4B–D, respectively). Importantly, the diversity indices were calculated based on the proportional data; therefore, they do not account for the fact that the GoodBiome™ Foods increased the total number of microbial cells.

**Figure 4:**
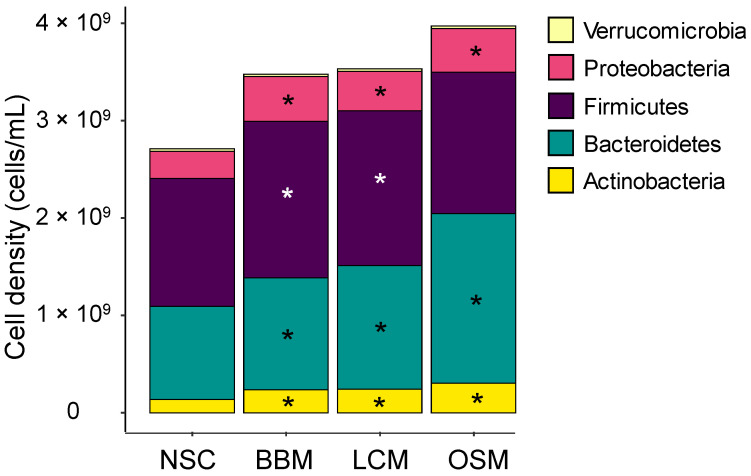
**GoodBiome™ Foods exerted significant impact on microbial composition at phylum level.** Samples were collected 30 h after the colonic incubations were initiated. Data were expressed as average absolute levels (cells/mL) of each phylum across simulations for four individual donors (n = 3 per donor). The statistical significance of the potential treatment effects within each comparison was determined via Benjamani–Hochberg post hoc testing. Significant changes (*p*_adjusted_ < 0.05) were indicated with asterisks.

After 30 h incubation, all three GoodBiome™ Foods significantly increased Actinobacteria, Bacteroidetes (particularly strong for OSM), and Proteobacteria, with BBM and LCM test products additionally increasing the Firmicutes phylum (Figure 4).

At the family level, each test product increased the abundance of many families relative to the unsupplemented control (NSC) (Figure S5). Increases in *Bifidobacteriaceae* (+1.22/1.24/1.42 log2fold change vs. NSC for BBM/LCM/OSM, respectively; *p*_adjusted_ < 0.001 for all treatments), *Prevotellaceae*, *Clostridiaceae*, and *Lachnospiraceae* abundance were common for all three test products. In addition, product-specific effects were observed; while OSM more strongly stimulated several families belonging to Bacteroidetes, including *Bacteroidaceae*/*Bacteroidales_u_f*/*Tannerellaceae* (+1.07/1.05/1.33 log2fold change vs. NSC for OSM (*p*_adjusted_ < 0.001 for all three families), in contrast to +0.48/0.48/0.11 for BBM (*p*_adjusted_ = 0.001/0.014/0.179) and +0.57/0.43/0.71 for LCM (*p*_adjusted_ = 0.007/0.034/<0.001)), BBM specifically stimulated *Peptococcaceae*, while LCM was the only product that significantly increased *Erysipelotrichaceae*.

**Figure 5:**
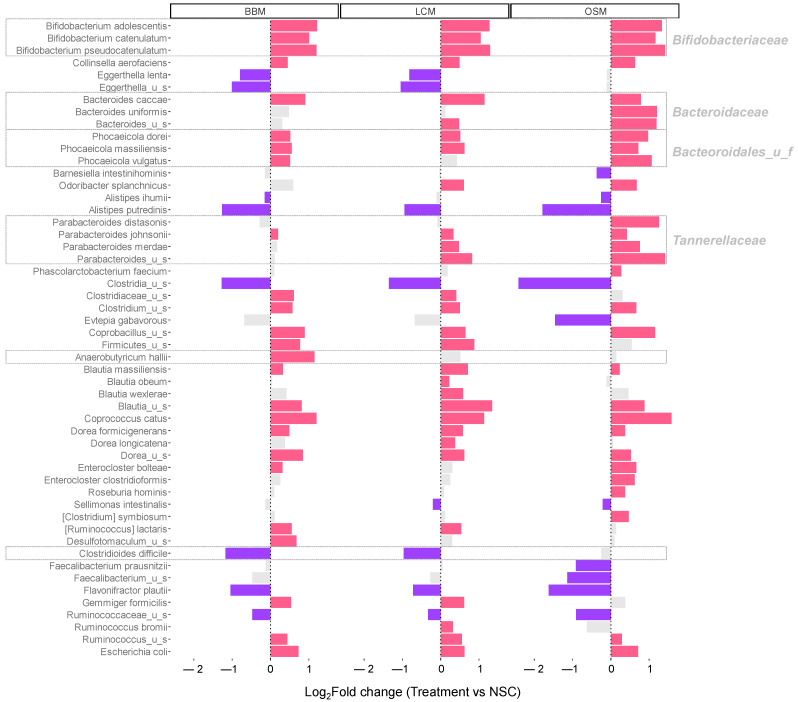
**GoodBiome™ Foods exerted significant impact on microbial composition at species level.** The bar charts were generated for species that were significantly (FDR = 0.05) affected by any of the treatments at 30 h, expressed as log2fold change (treatment/NSC), averaged across four human adults (n = 3 per donor). Purple and red bars indicated significant/consistent decreases and increases, respectively. Notable health- or disease-related taxa are highlighted in a gray box.

Changes in abundance at the species level are shown in Figure 5. All GoodBiome™ Foods exerted potent bifidogenic effects, i.e., they specifically increased several members in the family *Bifidobacteriaceae* including *B. catenulatum*, *B. adolescentis*, and *B. pseudocatenulatum*. Similarly, multiple species within the phylum Firmicutes, including *Clostridium_u_s*, *Coprobacillus_u_s*, *Coprococcus catus*, and *Dorea formicigenerans* were also significantly stimulated by all three GoodBiome™ Foods. 

GoodBiome™ Foods also exhibited product-specific effects. First, OSM exhibited markedly stronger specificity towards *Bacteroidaceae* members (including *Bacteroides caccae*, *Bacteroides uniformis)*, *Bacteroidales_u_f* members (including *Phocaeicola dorei*, *Phocaeicola massiliensis*, *Phocaeicola johnsonii*), *Tannerellaceae* members (including *Parabacteroides distasonis*, *Parabacteroides johnsonii*, *Parabacteroides merdae*), and *Phascolarctobacterium faecium*. Within the phylum Firmicutes, BBM was the only test product that significantly increased *Anaerobutyricum hallii* (+1.15 log2fold change vs. NSC for BBM (*p*_adjusted_ = 0.004), in contrast to +0.52 for LCM (*p*_adjusted_ = 0.073) and +0.14 for OSM (*p*_adjusted_ = 0.572)), while LCM and OSM specifically increased *Blautia obeum*/*Blautia wexlerae* and *Enterocloster clostridioformis*/*Roseburia hominis*, respectively.

**Figure 6:**
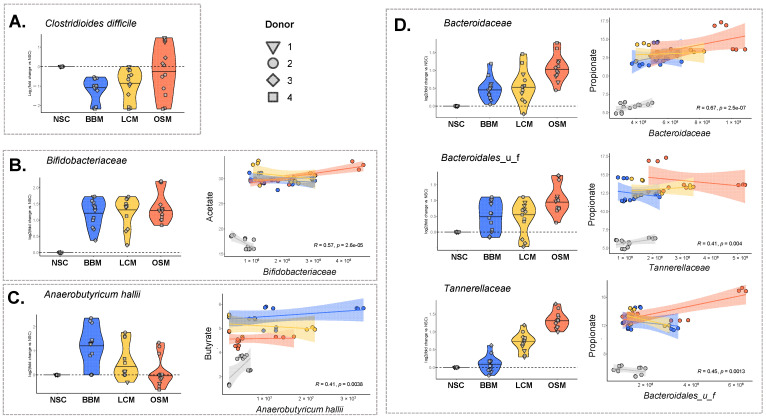
**The GoodBiome™ Foods exerted significant impact on taxa that are potentially relevant for human health.** Violin plots, expressed as log2fold change (treatment/NSC), were presented for four individual human adults (n = 3). The data were presented for (**A**) *Clostridiodes difficile* (**B**) *Bifidobacteriaceae*, (**C**) *Anaerobutyricum hallii*, (**D**) *Bacteroidaceae*, *Bacteroidales_u_f*, and *Tannerellaceae*. For (**B**–**D**), Pearson correlation analysis demonstrated significant positive correlations (*p* < 0.05) between the absolute levels of these taxa (cells/mL) and the concentration (mM) of the most relevant SCFA related to these taxa, i.e., (**A**) acetate, (**B**) butyrate, and (**C**) propionate.

Interestingly, the abundance of the pathogenic species *Clostridioides difficile* was significantly decreased upon fermentation of BBM and LCM (−1.17/−0.97/−0.25 log2fold change vs. NSC for BBM/LCM/OSM (p_adjusted_ = 0.001/0.008/0.610)) (Figure 6A).

Significant positive correlations between the production of SCFA and the abundances of major SCFA producers were established, in line with the aforementioned product-specific treatment effects. The *Bifidobacteriaceae* family (contains acetate-producing species) correlated with acetate that markedly increased for all treatments. Further, *Anaerobutyricum hallii* (major butyrate producer) correlated positively with butyrate (Figure 6B,C). In line with the striking effect of BBM on butyrate, BBM indeed most potently increased *A. hallii.* Further, *Bacteroidaceae*, *Bacteroidales_u_f*, and *Tannerellaceae* (families containing microbes that produce propionate or precursors thereof (e.g., succinate)) correlated positively with propionate (Figure 6D). In line with the striking effects of OSM on propionate, OSM indeed most potently increased these families. 

### 3.4. Fermentation of GoodBiome™ Foods Boosted the Production of Health-Promoting Microbial Metabolites 

**Figure 7:**
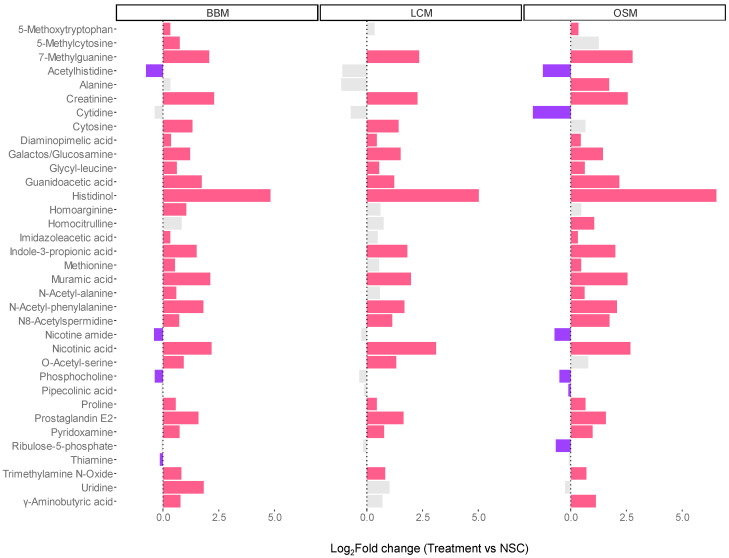
**The GoodBiome™ Foods exerted significant impact on the production of microbial metabolites, well beyond SCFA.** The bars were generated for metabolites that were significantly (FDR = 0.05) affected by any of the treatments, expressed as log2fold change (treatment/NSC), averaged across four human adults (n = 3 per test subject). Purple and red bars indicated significant decreases and increases, respectively.

The analysis of level 1- and 2a-annotated metabolites revealed that all GoodBiome Foods significantly boosted a wide range of metabolites, including a precursor (guanidinoacetic acid) and a breakdown product (creatinine) of creatine, 7-methylguanine, imidazoleacetic acid, indole-3-propionic acid, N8-acetylspermidine, and prostaglandin E2 (Figure 7). Two vitamin-related metabolites, nicotinic acid (vitamin B3) and pyridoxamine (vitamin B6), were also significantly increased with all three GoodBiome™ Foods. Further, product-specific effects were again observed. Most notably, the levels of 5-methoxytryptophan, imidazoleacetic acid, N-acetylalanine, and especially γ-Aminobutyric acid (GABA), a neurotransmitter playing an important role in the gut–brain axis, were only significantly elevated by BBM and OSM but not by LCM.

## 4. Discussion

Using the novel SIFR^®^ technology, which was recently shown to provide predictive insights for clinical findings [27], it was shown that all three GoodBiome™ Foods were well fermented by colonic microbes, as demonstrated by changes in pH, gas, SCFA levels, cell density, and levels of vitamins, essential amino acids, and health-related metabolites. Interestingly, this modulation was driven by specific bacterial species, depending on the exact ingredients that were incorporated in the GoodBiome™ Foods.

SCFAs, particularly acetate and propionate, were significantly increased with all GoodBiome™ Foods compared with NSC at all timepoints or at timepoints ≥ 24 h, respectively. Increased butyrate production was mainly observed with the BBM at timepoints ≥ 24 h and with LCM at 48 h. The production of these health-related SCFAs likely involved a broad range of gut microbes as incubation with GoodBiome™ Foods markedly altered microbial community composition versus NSC. First, all test products strongly increased *Bifidobacteriaceae* due to increases in a spectrum of species (*B. catenulatum*, *B. adolescentis*, and *B. pseudocatenulatum*). *Bifidobacterium* species are potent acetate and lactate producers [44] and have a well-established association with beneficial health effects [45]. They were likely boosted by GoodBiome™ Foods due to the inclusion of the prebiotic inulin in all three GoodBiome™ Foods, as inulin is a potent bifidogenic substrate [46]. GoodBiome™ Foods also increased the *Prevotellaceae*, *Enterobacteriaceae*, *Lachnospiraceae*, *Clostridiaceae*, *Bacteroidales* (unidentified family), and *Bacteroidaceae* families. Many of these families are associated with SCFA production, including *Lachnospiraceae* which contain members capable of producing butyrate [47], and *Bacteroidales*/*Bacteroidaceae* members, which produce acetate and propionate [48]. The stimulation of such a broad range of SCFA-producing bacteria indicates potential health benefits with the fermentation of GoodBiome™ Foods.

Interestingly, several product-specific effects on SCFA and SCFA-producing taxa were noted. A first remarkable finding was that the butyrogenic potential of BBM related to a specific increase in Anaerobutyricum *hallii*, a major butyrate producer [49] that is specialized in fermenting erythritol [50], indeed a main ingredient of BBM (Appendix A). Secondly, the potent propionogenic potential of OSM related to strongly increased levels of Bacteroidetes, a phylum known to increase upon consumption of oats [51], indeed a main ingredient of OSM. OSM, for instance, strongly increased *Bacteroidaceae* and *Bacteroidales_u_f*, which strongly correlated with propionate levels, in line with the fact that they, respectively, contain propionogenic *Bacteroides* and *Phocaeicola* species [49]. OSM also most strongly increased *Tannerellaceae*, which again correlated with propionate levels, in line with the fact that this family contains *Parabacteroides* species that are known to produce succinate [52], which can be converted to propionate by other species such as *Phascolarctobacterium faecium*, another species specifically stimulated by OSM. A third product-specific effect was an increase in members of the *Erysipelotrichaceae* family specific to the LCM product. The *Erysipelotrichaceae* family has been associated with a protective effect against colon cancer [53], and their abundance is reduced in patients with multiple sclerosis [54], new onset and recurrent Crohn’s disease [55,56], inflammatory bowel disease [57], and in children with autism spectrum disorder [58]. The reduced abundance in children with autism spectrum disorder is also associated with a reduction in butyrate, as *Erysipelotrichaceae* are butyrate producers [58]. Overall, the addition of specific ingredients to complex foods thus elicits the specific stimulation of SCFA-producing gut microbes, potentially eliciting specific health benefits. The present ex vivo study demonstrated a potent stimulation of *Bifidobacteriaceae* and *Bacteroidaceae* by GoodBiome™ Foods. This contrasts with findings from a recent in vitro M-SHIME^®^ study [59], which reported that GoodBiome™ Foods led to a bloom of *Enterobacteriaceae* and a stark decrease in *Bacteroidaceae* and especially *Bifidobacteriaceae.* The discrepancy results from fundamental differences in study designs. First, in the present SIFR^®^ study, the microbial community structure of the in vivo microbiota is maintained, and is thus an ex vivo simulation of the microbiota (amongst others, critical aspects relate to the use of an optimized nutritional medium and maintenance of anaerobicity, as elaborated before [27]), whereas the previous study used an in vitro technology, known to alter microbiota composition [60]. Secondly, the SIFR^®^ study incorporated an advanced simulation of upper gastro-intestinal digestion and absorption [28]. In absence of a parallel control in the previous in vitro study, the bloom in *Enterobacteriaceae* upon the administration of digested GoodBiome™ Foods likely reflects non-product related changes that could occur upon the in vitro digestion/absorption of complex food matrices. The SIFR^®^ protocol has been elaborated to integrate the optimal removal of oxygen, bile acids, and digestion products (e.g., amino acids), so as to avoid the introduction of bias that could lead to, e.g., *Enterobacteriaceae* bloom [61,62,63]. The combination of a biorelevant, ex vivo microbiological simulation with a physiologically relevant integration of digestion/absorption supports the validity of the present results.

GoodBiome™ Foods also decreased several species (e.g., *Flavonifractor plautii* and *Alistipes putredinis*), further suggesting the selective utilization of GoodBiome™ Foods by host microorganisms, a key feature in order to be classified as a prebiotic [64]. A finding of particular interest was that OSM/BBM suppressed the pathogenic species *Clostridioides difficile.* This might relate to the inclusion of the probiotic *B. subtilis* HU58™ in GoodBiome™ Foods. Indeed, HU58™ has been shown to possess a protein that is involved in an iron acquisition system, similar to the one *C. difficile* possesses [65], and may thus compete with *C. difficile* for iron acquisition. Such antipathogenic effects of HU58™-containing products were recently demonstrated in a mice model for *C. difficile* infection [66]. As a critical note, while other studies have already revealed a high survival of *B. subtilis* HU58™ along a simulated upper gastrointestinal passage compared to other probiotics (*Bifidobacterium longum* BB536 and *Lactobacillus acidophilus* DDS-1) [67], it would be interesting to further confirm the survival of *B. subtilis* HU58™ along the simulated upper gastrointestinal passage when ingested as part of GoodBiome™ Foods to further corroborate the potential contribution of *B. subtilis* HU58™ in the gut microbiome modulation by GoodBiome™ Foods.

In contrast to the other SCFA (acetate, propionate, and butyrate) levels, valerate, a less abundantly produced metabolite, decreased upon supplementation of GoodBiome™ Foods. While valerate is less studied than the other SCFAs, it has also been demonstrated to decrease the growth of cancer cells [68] and to exert antipathogenic effects against *C. difficile* [69]. Given that valerate levels were lower in study arms where *C. difficile* was inhibited, valerate did not contribute to such antipathogenic effects during the current study. Future studies are required to elucidate which gut microbes are involved in the production of valerate and how it impacts host health. Finally, all GoodBiome™ Foods significantly boosted levels of vitamins B3 and B6. 3-indolepropionic acid levels were significantly increased as well. This is a potent neuroprotective antioxidant produced from tryptophan which has recently been linked to the maintenance of intestinal epithelium homeostasis and a reduction in plasma endotoxin levels in rats [70]. The levels of essential amino acids (leucine, methionine, and phenylalanine) were also increased along with 4-guanidinobutyric acid (GABA), which is a major inhibitory neurotransmitter having relaxation, anti-anxiety, and anti-convulsive effects [71]. This increase was strongest with the OSM product, consistent with the fact that the GABA was most likely produced by *Bacteroides* and *Parabacteroides* species [72], which were most abundant with OSM fermentation. Other notable metabolites that increased with all GoodBiome™ Foods were 5-methoxytryptophan, an endothelial factor with anti-inflammatory properties [73]; muramic acid, a key component of peptidoglycan; sugar levels, which likely reflect the presence of residual sugars of the oligo- and polysaccharides present in the test products; and 3-(4-hydroxyphenyl)propionic acid, a product of flavonoid metabolism [74].

## 5. Conclusions

Our findings stress the need to apply physiologically relevant digestion/absorption procedures prior to studying the microbiome-modulating potential of complex foods using a technology that maintains the in vivo microbiota community. In doing so, this study demonstrated how the incorporation of specific ingredients in GoodBiome™ Foods specifically altered microbial metabolite production and composition, amongst others, including (i) inulin~acetate via *Bifidobacterium* species (all three GoodBiome™ Foods), (ii) erythritol~butyrate via *A. hallii* (BBM), and (iii) oat fiber~propionate by Bacteroidetes members (OSM). The application of metabolomics revealed that functional muffins also impacted metabolite production well beyond the traditionally studied SCFAs. It would be interesting to study the potential health benefits that could follow from such beneficial gut microbiome modulation of the currently tested products.

Further, future product development could focus on assessing potential benefits of replacing inulin with prebiotics with higher selectivity towards specific gut microbes (optimally including the probiotics included in GoodBiome™ Foods such as *B. subtilis* HU58). While inulin has well described health benefits [75], it is associated with high gas production and lower tolerability (due to side effects as flatulence and bloating) [76]. Moreover, inulin is shown to have a rather low selectivity in terms of how it impacts the gut microbiome (inulin can be fermented by different gut microbes depending on an individual’s microbiome) [32]. The inclusion of more selective precision prebiotics in GoodBiome™ Foods could thus potentially more strongly stimulate the growth of co-administered probiotics, enhancing tolerability, while also having a higher predictivity of gut microbiome modulation and subsequent health benefits.

## Data Availability

The datasets generated during and/or analyzed during the current study are available from the corresponding author upon reasonable request.

## Supplementary Materials

The following supporting information can be downloaded at https://www.mdpi.com/article/10.3390/metabo14090497/s1, Figure S1: The fecal microbiota covered clinically relevant interpersonal differences; Figure S2: GoodBiome™ Foods exerted marked effects on key fermentation parameters over time; Figure S3: GoodBiome™ Foods exerted marked effects on the production of short-chain fatty acids; Figure S4: GoodBiome™ Foods exerted marked effects on cell growth and microbial diversity; Figure S5: GoodBiome™ Foods affected different bacterial families across different donors.

## Appendix A

Test product composition.
GoodBiome™ Foods Lemon Chia Muffin (Microbiome Labs)
Inulin powder, white sorghum flour, pea protein concentrate, flaxseed, soluble tapioca fiber, almond protein concentrated, black chia seeds, cane sugar, almond meal, sacha inchi protein concentrate; contains 2% or less of each of the following: leavening (monocalcium phosphate, sodium bicarbonate), xylooligosaccharides, lemon, tapioca starch, sea salt, orange powder, banana powder, papaya powder, shiitake mushroom powder, monk fruit extract, *Bacillus subtilis* HU58™, natural flavor.
GoodBiome™ Foods Berry Blast Muffin (Microbiome Labs)
Inulin powder, white sorghum flour, pea protein concentrate, flaxseed meal, erythritol, almond protein concentrate, almond meal, quinoa flour, coconut sugar, sacha inchi protein concentrate; contains 2% or less of each of the following: date powder, blueberry powder, strawberry powder, soluble tapioca fiber, cranberry powder, beet powder, leavening (monocalcium phosphate, sodium bicarbonate), xylooligosaccharides, monk fruit extract, orange powder, banana powder, papaya powder, shiitake mushroom powder, sea salt, tapioca starch, natural flavor, *Bacillus subtilis* HU58™.
GoodBiome™ Foods Oat Spice Mookie (Microbiome Labs)
Oats, almond meal, inulin powder, pea protein concentrate, flaxseed, soluble tapioca fiber, chocolate chips (cane sugar, cocoa liquor, cocoa butter), cane sugar; contains 2% or less of the following: leavening (monocalcium phosphate, sodium bicarbonate), xylooligosaccharides, fenugreek seed, anise, molasses, sea salt, orange powder, banana powder, papaya powder, shiitake mushroom powder, monk fruit extract, *Bacillus subtilis* HU58™.
