# Supplementary material for: Functional Muffins Exert Bifidogenic Effects along with Highly Product-Specific Effects on the Human Gut Microbiota Ex Vivo
Source: Metabolites. 2024 Sep 14;14(9):497. doi: 10.3390/metabo14090497 (PMC11433953; doi:10.3390/metabo14090497)
Supplement: Supplementary file 1 [file metabolites-14-00497-s001.zip › metabolites-3176355-supplementary.pdf]

metabolites

MDPI

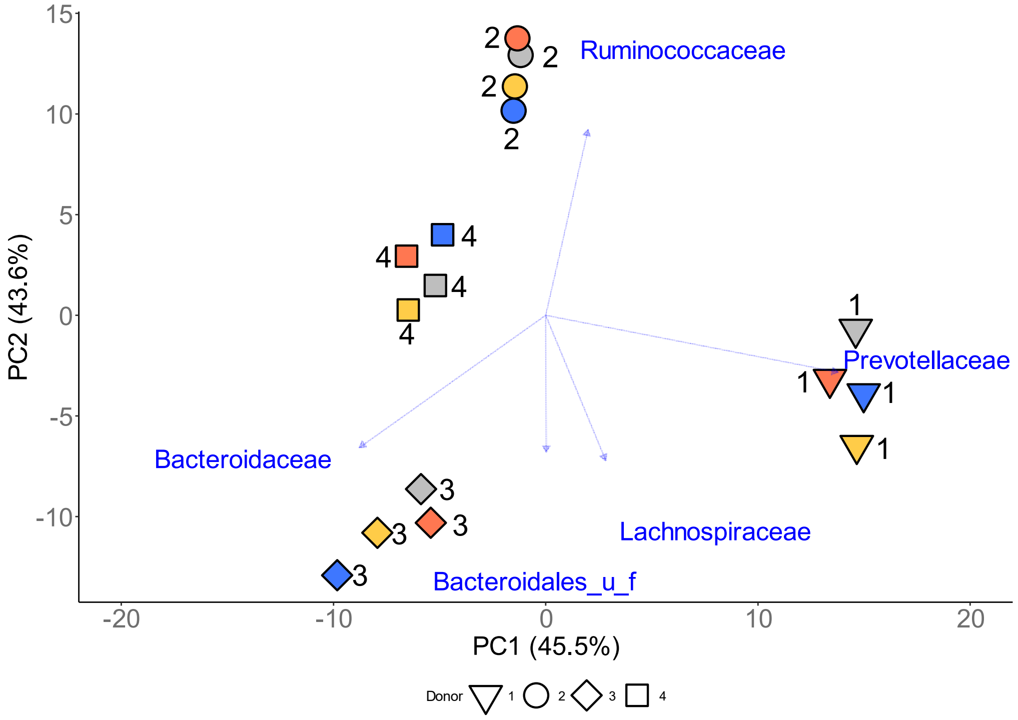

Figure S1. The fecal microbiota covered clinically relevant interpersonal differences. Principal component analysis (PCA) summarizing the microbial community composition of the four human adults that provided a fecal donation for the current SIFR® study. The PCA was calculated based on the (centered) abundances (%) of the microbial families, as quantified via shallow shotgun sequencing.

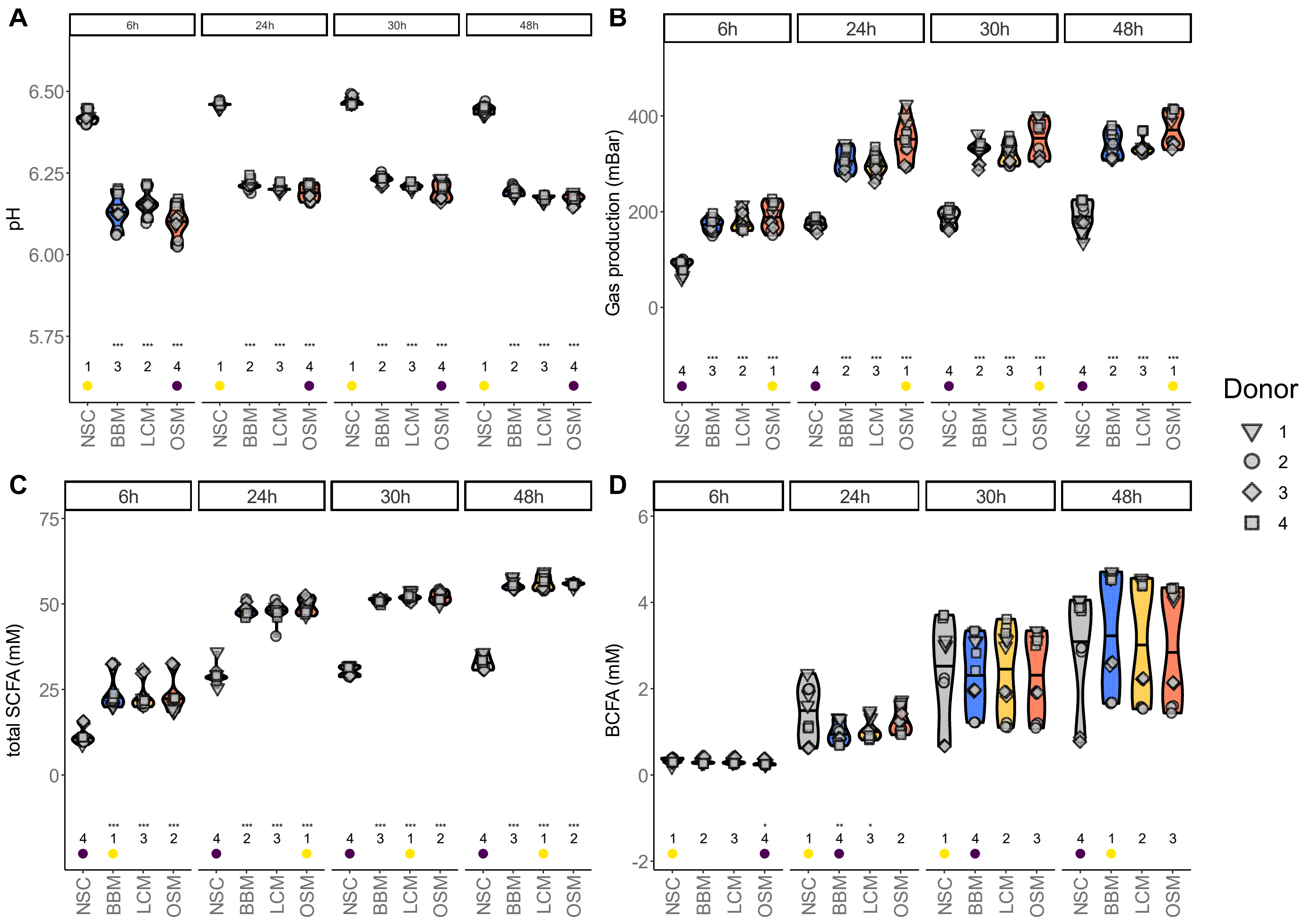

Figure S2. GoodBiome™ Foods exerted marked effects on key fermentation parameters over time. The effects on (**A**) pH, (**B**) gas production, (**C**) total SCFA and (**D**) bCFA were compared for the GoodBiome™ Foods versus an unsupplemented control (NSC) at 6h, 24h, 30h, and 48h after initiation of colonic incubation. The data is presented using values for individual donors. Statistical significance of the treatment effects for the test products vs. NSC within each time point are indicated by * ($0.01 < p_{adjusted} < 0.05$), ** ($0.001 < p_{adjusted} < 0.01$) or *** ($p_{adjusted} < 0.001$). The ranks of the average values per treatment are indicated at the bottom of the figure, with the lowest average being indicated in purple, and the highest value in yellow.

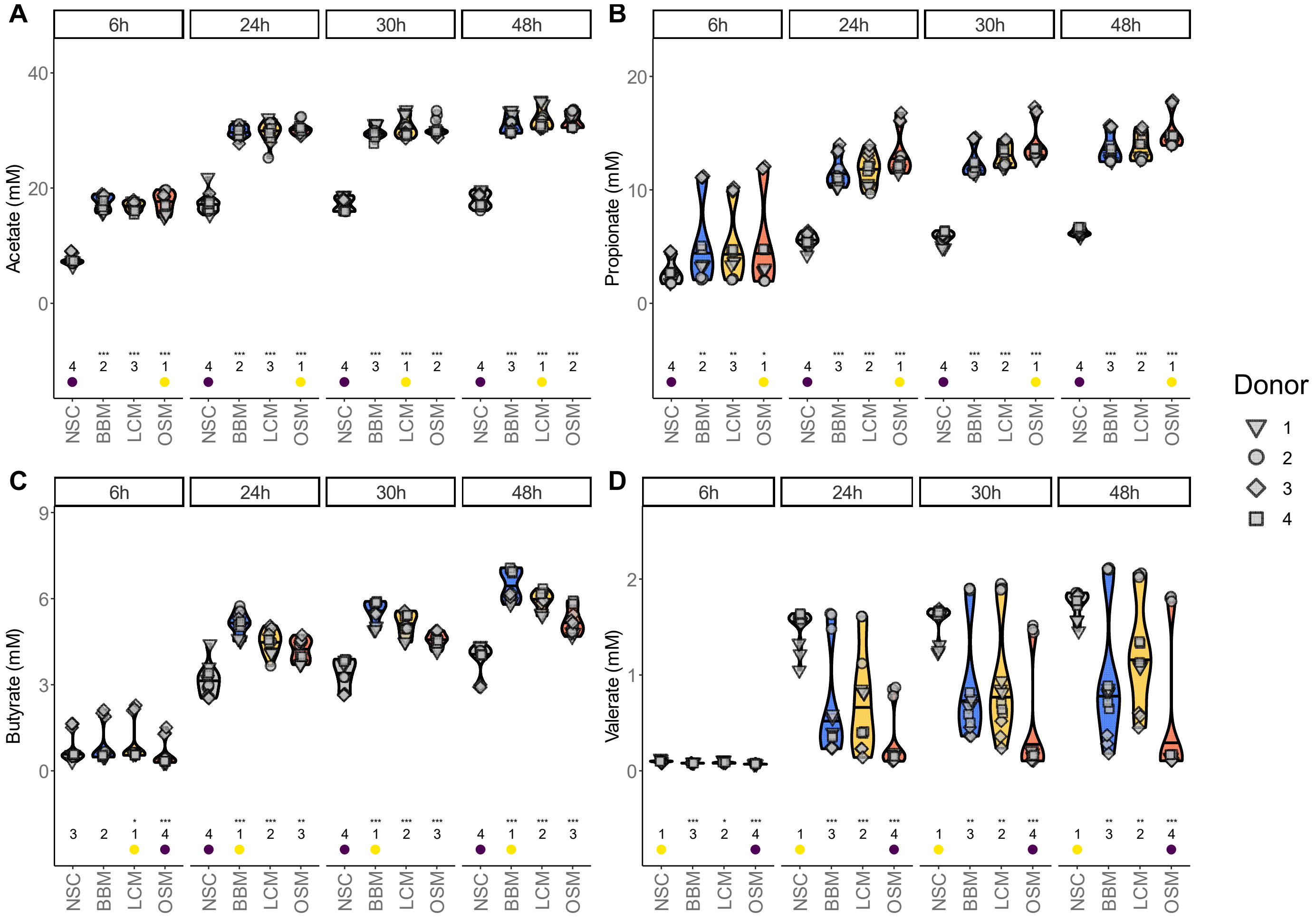

Figure S3. GoodBiome™ Foods exerted marked effects on the production of short-chain fatty acids. The effects on (**A**) acetate, (**B**) propionate, (**C**) butyrate and (**D**) valerate were compared for the GoodBiome™ Foods versus an unsupplemented control (NSC) at 6h, 24h, 30h, and 48h after initiation of colonic incubation. The data is presented using values for individual donors. Statistical significance of the treatment effects for the test products *vs*. NSC within each time point are indicated by * ($0.01 < p_{adjusted} < 0.05$), ** ($0.001 < p_{adjusted} < 0.01$) or *** ($p_{adjusted} < 0.001$). The ranks of the average values per treatment are indicated at the bottom of the figure, with the lowest average being indicated in purple, and the highest value in yellow.

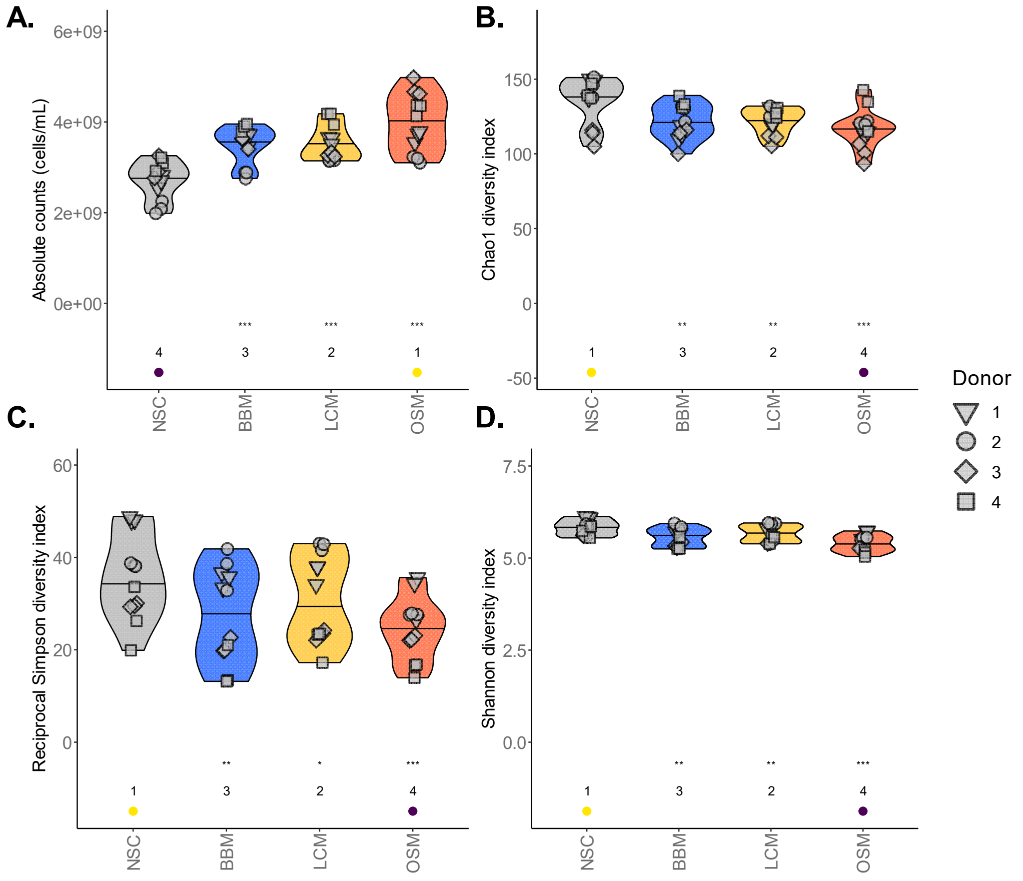

Figure S4. GoodBiome™ Foods exerted marked effects on cell growth and microbial diversity. The effect on (**A**) total cell counts, (**B**) Chao1 diversity index, (**C**) reciprocal Simpson index and (**D**) Shannon diversity index was compared for the GoodBiome™ Foods versus an unsupplemented control (NSC) at 30h after initiation of colonic incubation. The data is presented using values for individual donors. Statistical significance of the treatment effects for the test products *vs.* NSC within each time point are indicated by * ($0.01 < p_{adjusted} < 0.05$), ** ($0.001 < p_{adjusted} < 0.01$) or *** ($p_{adjusted} < 0.001$). The ranks of the average values per treatment are indicated at the bottom of the figure, with the lowest average being indicated in purple, and the highest value in yellow.

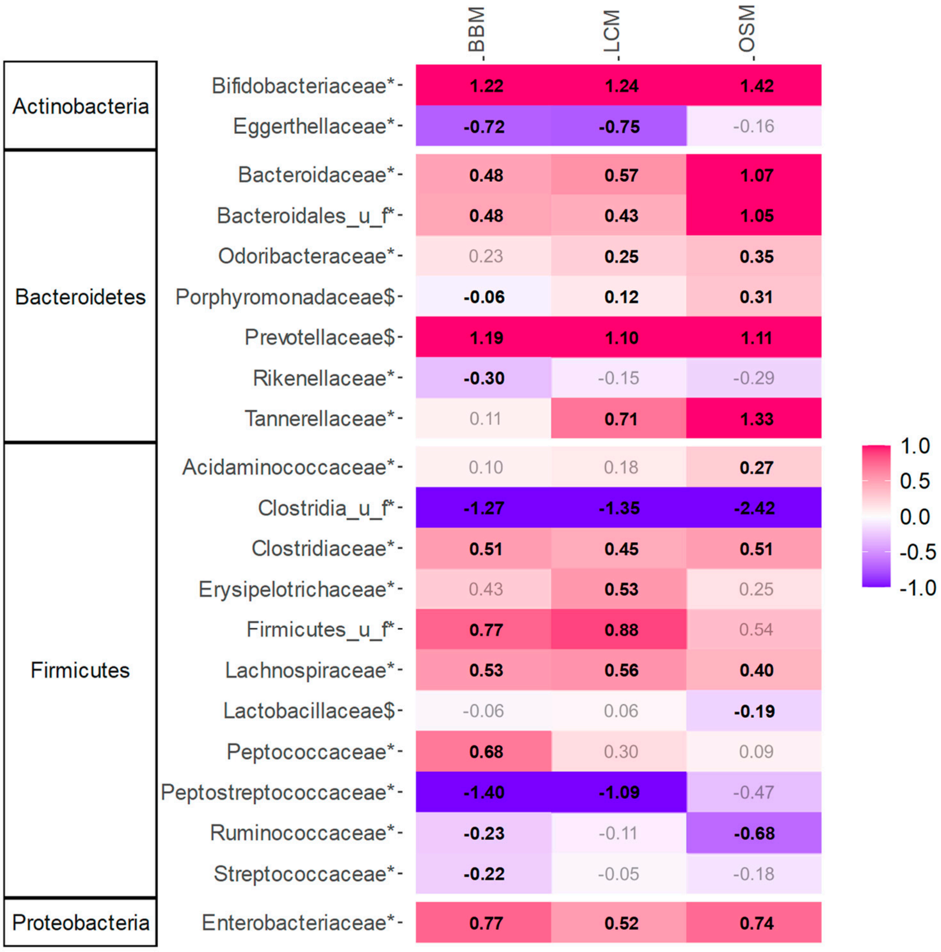

Figure S5. GoodBiome™ Foods affected different bacterial families across different donors. Heatmap was generated based on families that were significantly (FDR = 0.05) and non-significantly but consistently affected by the test products at 30 h, expressed as $\log_2$ foldchange (treatment/NSC), averaged over four human adults (n = 3 per donor). Asterisks (*) indicate families that exhibited significant changes upon treatment. Dollar signs ($) indicate families that exhibited consistent changes upon treatment. Numbers in bold indicate the treatments where significant or consistent changes compared to the NSC occurred. The corresponding families and phyla are indicated on the left.
